# Long-term prevalence of PTSD symptom in family members of severe COVID-19 patients: a serial follow-up study extending to 18 months after ICU discharge

**DOI:** 10.1186/s40560-024-00765-9

**Published:** 2024-12-18

**Authors:** Nobuyuki Nosaka, Ayako Noguchi, Takashi Takeuchi, Kenji Wakabayashi

**Affiliations:** 1 Intensive Care Unit, Institute of Science Tokyo Hospital, 1-5-45 Yushima Bunkyo-Ku, Tokyo, 113-8510 Japan; 2 Department of Intensive Care Medicine, Graduate School of Medical and Dental Sciences, Institute of Science Tokyo, 1-5-45 Yushima Bunkyo-Ku, Tokyo, 113-8510 Japan; 3 Department of Disaster and Critical Care Nursing, Track of Nursing Innovation Science, Graduate School of Health Care Sciences, Institute of Science Tokyo, 1-5-45 Yushima Bunkyo-Ku, Tokyo, 113-8510 Japan; 4 Department of Psychiatry and Behavioral Neurosciences, Graduate School of Medical and Dental Sciences, Institute of Science Tokyo, 1-5-45 Yushima Bunkyo-Ku, Tokyo, 113-8510 Japan

**Keywords:** COVID 19, Health Related quality of life (HRQOL), Intensive care unit (ICU), Post Intensive care syndrome, Family (PICSF), Post Traumatic stress disorder (PTSD)

## Abstract

**Background:**

Experiencing a loved one's stay in the intensive care unit (ICU) can profoundly affect families, often leading to post-intensive care syndrome-family (PICS-F), a condition particularly exacerbated during the COVID-19 pandemic. While PICS-F significantly impacts the mental health of families of ICU patients, especially in the context of COVID-19, the long-term effects beyond 12 months remain understudied. This study aims to explore the prevalence of PTSD-related symptoms and health-related quality of life (HRQOL) in family members up to 18 months after ICU discharge.

**Methods:**

This prospective study, conducted in a tertiary university hospital in Tokyo, enrolled family members of severe COVID-19 ICU patients (July 2020 to June 2022 with final follow-up ending in December 2023). The primary outcome was family member symptoms of PTSD at 6, 12 and 18 months after ICU discharge, measured by the Impact of Events Scale-Revised (presence of PTSD symptoms defined by score > 24). Secondary outcomes were family member symptoms of anxiety and depression, sleep disorders, and health-related quality of life (HRQOL) at the same timepoint.

**Results:**

Among 97 enrolled family members, 68 participated. At least one PTSD-related symptom was reported by 26% of family members, persisting over 18 months post-discharge (16% at 6 months, 23% at 12 months, and 25% at 18 months). A subgroup (15%) exhibited delayed-onset PTSD symptoms. Family members with PTSD-related symptoms reported lower HRQOL, especially in mental and social components.

**Conclusions:**

The study underscores the importance of long-term support for family members post-ICU discharge, given the sustained prevalence of PTSD-related symptoms among family members of severe COVID-19 patients.

## Background

Experiencing a loved one's stay in the intensive care unit (ICU) deeply affects the family on an emotional level [1]. This phenomenon, known as post-intensive care syndrome-family (PICS-F), encompasses new or exacerbated mental challenges during or after the patient's ICU stay [2–5]. COVID-19 has particularly impacted the families of patients receiving ICU care for several reasons including emotional distress, communication challenges with healthcare providers, limited hospital visitation, financial strain, and health risks of contracting COVID-19 [6–9]. Recent research has brought to light the enduring effects over months on these families, underscoring the importance of social support not only during the patient's hospitalization but also after the patient is discharged [10–19]. With the likelihood of similar pandemics in the future [20], readiness and preventative measures for diverse healthcare challenges remain essential in mitigating the complex repercussions of potential outbreaks. Insights into the long-term effects over the years on family members of COVID-19 patients can play a vital role in this preparedness, aiding in the development of effective countermeasures for PICS-F even in non-pandemic times. While it is known that PICS-F can affect mental health for years, studies with long follow-up of COVID-19-related PICS-F for more than 12 months have been limited. Accordingly, this study explores the prevalence of post-traumatic stress disorder (PTSD)-related symptoms in the remote phase following a patient's ICU discharge, tracking the mental health and health-related quality of life (HRQOL) of the patient's family members for up to 18 months after the patient’s ICU discharge.

## Methods

### Participants

This study was conducted in a tertiary university hospital in Tokyo, Japan. We included one ‘key family member’ per adult patient admitted to the ICU between July 2020 and June 2022 for severe COVID-19. The involved family member was the adult with whom we had mainly communicated during the patient’s ICU stay. In addition, family members of patients who passed away in the ICU were also invited to participate.

### Study procedures

**Fig. 1:**
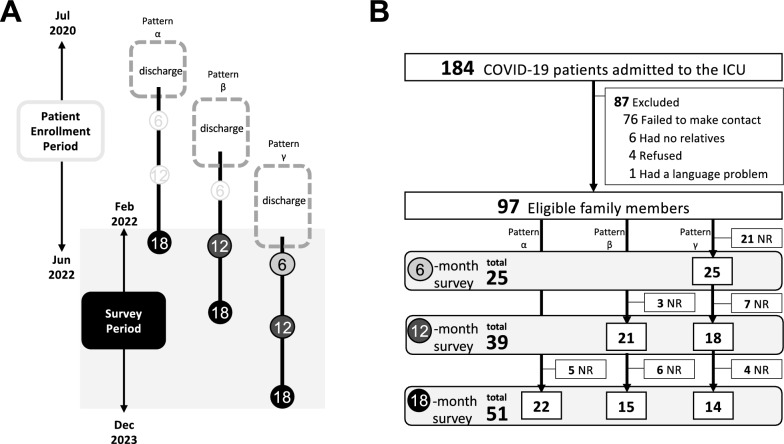
Survey strategy (**A**) and selection of family members for the study (**B**). NR: no response

In response to the surge of the COVID-19 pandemic, we set up the new pandemic ICU to accept severe COVID-19 patients in April 2020. We have flexibly controlled the number of operating beds for the COVID-19 ICU and non-COVID-19 ICUs, corresponding to the number of COVID-19 patients in Tokyo. We have had up to 22 beds in the ICU for COVID-19 patients during the study inclusion period. We started to recruit eligible family members in February 2022. Study candidates were initially contacted via phone for participation in a questionnaire survey one month before the earliest survey timepoint, which was set at 6, 12, or 18 months after the patients’ ICU discharge. Once consent was obtained on the phone, the questionnaires were mailed to the family members at the predetermined timepoints described above. Moreover, for the family members who responded to the survey at the 6- or 12-month timepoints after the patient’s discharge, we mailed additional follow-up surveys at 12 and/or 18 months after the patient’s discharge, requesting their responses to the same questionnaires. Because we began this study more than a year after the epidemic began, some study participants could only be surveyed once at 18 months, depending on the timing of the corresponding patient’s ICU discharge, while others were surveyed two or three times for follow-up (Fig. 1A). Subsequent follow-up surveys were mailed at a set timepoint without contacting the family. No further surveys were mailed to family members who did not return the initial or secondary survey.

### Questionnaire

Participants were asked to respond to the following four types of questionnaires in each survey; the impact of event scale-revised (IES-R) [21], the hospital anxiety and depression scale (HADS) [22], the Pittsburgh sleep quality index (PSQI) [23], and the 12-item short-form health survey (SF-12) [24]. It should be noted that we used the Japanese version of SF-12, which calculates scores for three components [25]: the physical health component summary (PCS), the mental health component summary (MCS), and the role-social component summary (RCS). While the original English version of SF-12 allows for the calculation of two component summary scores (PCS and MCS) from the eight subscales, this approach is not recommended for Japanese people due to differences in factor structures. Instead, a three-component scoring method was developed by incorporating role/social dimensions (RCS) into PCS and MCS, based on findings from a large-scale population study in Japan. We used these questionnaires, because they have been used in previous studies on PICS and recommended in a recent review [26]. Participants were asked to provide the date of each survey response. In addition, they were asked to fill out the individual data including age, sex, and relationship to the corresponding patient in the initial survey.

### Patient data collection

We extracted the following information about the corresponding patients from the medical records in a retrospective manner: age, sex, clinical interventions including mechanical ventilation, tracheostomy, and extracorporeal membrane oxygenation (ECMO) therapy, and clinical outcomes including ICU mortality and length of stay (LOS) in the ICU. We also calculated the number of days between the patient’s discharge from the ICU and the answer date by the family member.

### Outcomes

The study population was the family members of patients hospitalized in severe COVID-19 ICU. The primary outcome was the prevalence of PTSD-related symptoms determined by IES-R among family members in the remote phase after patients’ ICU discharge.

### Data analysis

Continuous variables are presented as medians and interquartile ranges (IQRs), while categorical variables are expressed as counts and percentages. The comparison between groups involved the application of the student’s *t* test or Wilcoxon rank-sum test where appropriate for quantitative variables and the Fisher exact test for qualitative variables. The IES-R, the anxiety subscale of the HADS (HADS-A), the depression subscale of the HADS (HADS-D), and the PSQI were dichotomized using specific thresholds to identify the presence of PTSD (IES-R ≧ 25) [21], anxiety (HADS-A ≧ 8), depression (HADS-D ≧ 8) [27], and sleep disorder-related symptoms (PSQI ≧ 8) [28].

## Results

### Participant characteristics

A summary of participant recruitment and follow-up is provided in Fig. 1B. During the enrolment period, 184 patients were admitted to the COVID-19 ICU. Of 178 patients' families approached, 97 were enrolled with verbal consent over the phone. The primary reason for non-enrolment was the failure of the family to respond to the telephone invitation to participate in the study (*n* = 76 [87%]).

Of the enrolled family members, 68 (70%) participated in this study. In terms of the family member factors, the median (IQR) age was 55 (49–70) years; 77% were women, and 44% were the patient’s spouses. Regarding the patient-related factors, the mean age was 66 (53–76) years; 18% were female, and 90% underwent mechanical ventilator management with a median of 10 (6–15) days; patients had spent a median of 11 (7–20) days in the ICU; 9 (13%) patients died in the ICU (Table 1).

**Table 1:** Characteristics of family members and patients: comparison between with and without PTSD-related symptoms

Factor	Group	Total *n* = 68	With PTSD-related symptoms* n = 18 (26%)	Without PTSD-related symptoms *n* = 50 (74%)	p value
Family member factors					
Age (year), median [IQR]		55 [49, 70]	64 [50, 72]	54 [49, 67]	0.297
Female sex, No. (%)		52 (77)	17 (94)	35 (70)	0.051
Relationship (%)					
	Child	18 (27)	1 ( 6)	17 (34)	0.038
	Mother	8 (12)	2 (11)	6 (12)	
	Siblings	8 (12)	1 ( 6)	7 (14)	
	Spouse	30 (44)	13 (72)	17 (34)	
	Others	4 ( 6)	1 ( 6)	3 ( 6)	
Patient-related factors					
Age (year), median [IQR]		66[53, 76]	65 [55, 75]	67 [53, 76]	0.612
Female sex, No. (%)		12 (18)	2 (11)	10 (20)	0.494
Mechanical ventilation, No. (%)		61 (90)	17 (94)	44 (88)	0.666
Length of Mechanical ventilation, median [IQR]		10 [6, 15]	10 [6, 15]	10 [6, 15]	0.705
ECMO, No. (%)		4 ( 6)	2 (11)	2 ( 4)	0.284
Tracheostomy, No. (%)		15 (22)	5 (28)	10 (20)	0.519
Length of ICU stay (day), median [IQR]		11 [7, 20]	11 [7, 20]	11 [6, 18]	0.749
ICU mortality (%)		9 (13)	2 (11)	7 (14)	1

* identified positive at least one of multiple surveys

### Prevalence of PTSD-related symptoms in the remote phase after patient’s ICU discharge

Among the 68 participants, 18 family members (26%) had PTSD-related symptoms (IES-R ≧ 25) at least once in this study. There were no differences in family member and patient characteristics between the family members with or without PTSD-related symptoms, except for the relationship to the patient, as 72% of the family members with PTSD-related symptoms were spouses compared to 34% of those without the symptoms (p = 0.038) (Table 1). It is also reasonable to assert that there was a higher prevalence of females among family members with PTSD-related symptoms compared to those without symptoms (94% vs 70%, p = 0.051).

Of the 68 participants, 25, 39, and 51 responded to the 6-month, 12-month, and 18-month follow-up surveys, respectively (Fig. 1B). Table 2 describes the prevalence and characteristics of family members with or without PTSD-related symptoms at each survey point. At 6, 12, and 18 months after the patient’s ICU discharge, PTSD-related symptoms were experienced by 4/25 (16%), 9/39 (23%), and 13/51 (25%) family members, respectively. The family members with PTSD-related symptoms reported accompanied psychiatric symptoms, including anxiety and depression more commonly. They also reported lower health-related quality of life for MCS and RCS than those without PTSD-related symptoms. In particular, the prevalence of anxiety was significantly higher (100% vs 14% at 6 months, p = 0.001; 100% vs 13% at 12 months, p < 0.001; 77% vs 11% at 18 months, p < 0.001), and the RCS was significantly lower at all three survey points (28.40 vs 48.67 at 6 months, p = 0.009; 36.21 vs 50.05 at 12 months, p < 0.001; 42.18 vs 52.43 at 18 months, p < 0.001).

**Table 2:** Characteristics of family members: comparison between with and without PTSD-related symptoms across survey points

Factor	Group	6 months after Patient's Discharge	With PTSD- related symptoms *n* = 4	p value	12 months after Patient's Discharge	With PTSD- related symptoms *n* = 9	p value	18 months after Patient's Discharge	With PTSD- related symptoms *n* = 13	p value
		Without PTSD- related symptoms *n* = 21			Without PTSD- related symptoms *n* = 30			Without PTSD- related symptoms *n* = 38		
Assessment day, median [IQR]		210.0 [203.0, 222.0]	189.0 [184.0, 192.0]		378.5 [362.0, 394.5]	375.0 [352.0, 395.0]		555.5 [539.3, 579.5]	562.0 [548.0, 570.0]	
IES-R score, median [IQR]		5.0 [0.0, 10.0]	37.0 [32.0, 43.3]	0.002	10.0 [4.3, 15.0]	38.0 [35.0, 51.0]	< 0.001	3.5 [1.0, 11.8]	38.0 [32.0, 44.0]	< 0.001
Scores and Symptoms										
HADS anxiety subscale score, median [IQR]		4.0 [1.0, 5.0]	11.5 [10.5, 12.5]	0.002	2.0 [1.0, 5.0]	9.0 [8.0, 12.0]	< 0.001	1.0 [0.0, 3.8]	11.0 [9.0, 12.0]	< 0.001
Family members with anxiety- related symptoms, No. (%)		2 (10)	4 (100)	0.001	4 (13)	9 (100)	< 0.001	4 (11)	10 (77)	< 0.001
HADS depression subscale score, median [IQR]		4.0 [2.0, 5.0]	13.0 [11.3, 14.0]	0.005	4.5 [2.0, 6.5]	8.0 [6.0, 10.0]	0.009	3.0 [1.0, 6.0]	10.0 [9.0, 12.0]	< 0.001
Family members with depression- related symptoms, No. (%)		3 (14)	4 (100)	0.003	6 (20)	5 (56)	0.085	6 (16)	10 (77)	< 0.001
PSQI, median [IQR]		5.0 [3.0, 6.0]	8.0 [7.0, 13.5]	0.053	5.0 [2.0, 7.0]	9.0 [7.0, 13.0]	0.004	5.0 [2.0, 7.0]	8.0 [6.0, 9.0]	0.029
Family members with sleep disorder- related symptoms, No. (%)		5 (24)	3 (75)	0.081	8 (27)	5 (56)	0.129	11 (29)	7 (54)	0.177
SF-12, mean (SD)										
	MCS	54.7 (7.2)	44.2 (7.9)	0.027	53.4 (8.1)	48.8 (7.2)	0.160	55.4 (7.6)	49.4 (6.0)	0.014
	PCS	52.9 (7.6)	54.5 (17.7)	0.777	51.5 (7.6)	54.0 (9.9)	0.433	48.5 (8.9)	46.3 (13.3)	0.503
	RCS	48.7 (11.4)	28.4 (12.8)	0.009	50.1 (8.6)	36.2 (9.4)	< 0.001	52.4 (6.7)	42.2 (6.1)	< 0.001

### Tracking individual surveys

**Fig. 2:**
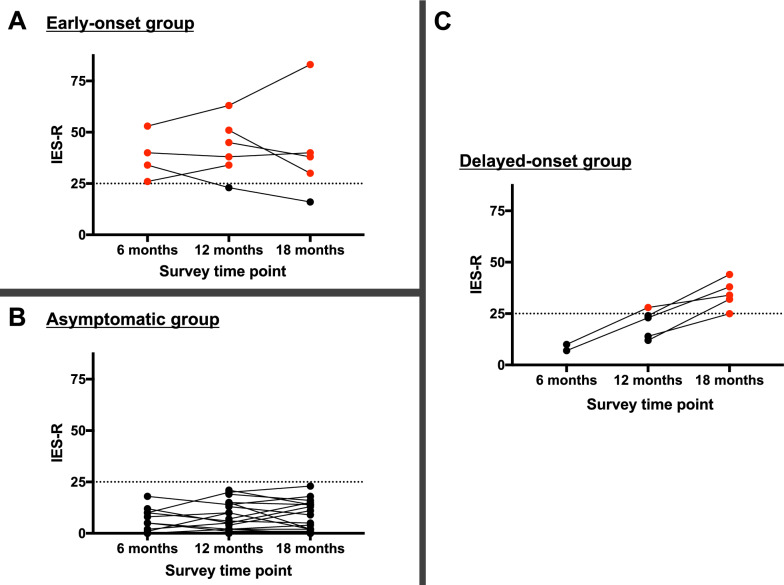
Time plot of IES-R scores in each patient according to symptom onset period. There were 6, 5, and 22 family members in the early-onset, the delayed-onset, and the asymptomatic group, respectively

Of the 68 participants, 33 family members responded to two (*n* = 19) or three (*n* = 14) surveys. Figure 2 shows the change in IES-R scores over time between surveys of these family members. Among them, 6 (18%) presented PTSD-related symptoms from their initial survey (Fig. 2A). Five of the six family members (83%) kept IES-R high in the subsequent surveys. On the other hand, the remaining 27 (82%) were not symptomatic (IES-R < 25) in the initial survey (Fig. 2B, C). However, 5 of the 27 (19%, or 15% of the 33) turned out to be symptomatic with PTSD-related symptoms in the subsequent surveys after the patient’s discharge, and we named this group “the delayed-onset group” (Fig. 2C).

### Characteristics of “the Delayed-onset Group”

Subsequently, we compared the backgrounds of family members and corresponding patients, including the psychological symptoms in the initial survey between the asymptomatic group and the delayed-onset group (Table 3). The family members in the delayed-onset group were all spouses of the corresponding patients. Patient-related factors were all similar between groups. Although the score of IES-R was not significantly different, the scores of HADS-A and HADS-D were significantly higher in the delayed-onset group with more family members presenting symptoms of depression.

**Table 3:** Characteristics of family members of severe COVID-19 patients: asymptomatic group vs delayed-onset PTSD-related syndromes

Factor	Group	The asymptomatic group *n* = 22	The delayed-onset group *n* = 5	p value
Family member factors				
Age (year), median [IQR]		55 [50, 67]	68 [49., 73.]	0.399
Female sex, No. (%)		18 (82)	5 (100)	0.561
Relationship (%)				
	Child	7 (32)	0 (0)	0.055
	Mother	5 (23)	0 (0)	
	Siblings	3 (14)	0 (0)	
	Spouse	5 (23)	5 (100)	
	Others	2 ( 9)	0 (0)	
Patient-related factors				
Male sex, No. (%)		17 (77)	5 (100)	0.547
Age (year), median [IQR]		58 [50, 74]	72 [55, 74]	0.639
Mechanical ventilation, No. (%)		20 (91)	5 (100)	1
Length of Mechanical ventilation, median [IQR]		10 [6, 18]	9 [8, 14]	0.812
ECMO, No. (%)		2 ( 9)	0 (0)	1
Tracheostomy, No. (%)		4 (18)	1 (20)	1
Length of ICU stay (day), median [IQR]		12 [8, 21]	11 [8, 14]	0.639
ICU mortality (%)		4 (18)	0 (0)	0.561
Scores and Symptoms at the initial survey				
IES-R score, median [IQR]		6.0 [2.0, 12.8]	12.0 [10.0, 14.0]	0.117
HADS anxiety subscale score, median [IQR]		1.5 [1.0, 4.5]	6.0 [5.0, 9.0]	0.017
Family members with anxiety-related symptoms, No. (%)		2 (9)	2 (40)	0.144
HADS depression subscale score, median [IQR]		4.0 [2.0, 5.0]	8.0 [5.0, 8.0]	0.044
Family members with depression-related symptoms, No. (%)		2 (9)	3 (60)	0.030
PSQI, median [IQR]		5.0 [3.0, 7.5]	5.0 [5.0, 6.0]	0.943
Family members with sleep disorder-related symptoms, No. (%)		8 (36)	1 (20)	0.636
SF-12, mean (SD)				
	MCS	53.9 (7.2)	50.9 (6.8)	0.404
	PCS	51.0 (8.8)	49.4 (2.4)	0.707
	RCS	51.6 (10.1)	42.9 (14.5)	0.128

## Discussion

In this prospective study in a single ICU in Tokyo, we observed that a total of 26% of family members of severe COVID-19 patients were suffering from PTSD-related symptoms, often accompanied by other psychiatric disorders, with stable trends over time, even in the remote phase after patients’ discharge (16% at 6 month, 23% at 12 months, and 25% at 18 months). Prior studies have described that family members of COVID-19 patients who were admitted to the ICU presented psychological disorders including anxiety, depression, and PTSD with various but high prevalence (2–90%) at different phases (30 days–1 year) after the patient’s discharge [10–18]. This research expands on previous findings by conducting follow-ups with the same individual family members, revealing that those with PTSD-related symptoms continued to experience them over an extended period.

We found that the family members with PTSD-related symptoms significantly diminished mental and social HRQOL (MCS and RCS) compared to those without the symptoms. This observation might be due to a variety of factors surrounding the COVID-19 pandemic. The suspension of ICU visits might be challenging for the family members, which could amplify psychological distress [29]. The quarantine and isolation due to the patient's SARS-CoV-2 infection might impact the HRQOL of family members [30]. Shirasaki et al. [19] described in a prospective cohort study in a single ICU in Tokyo that living in the same house with the patient was significantly associated with lower development of psychiatric disorders in the family members, which in turn means that isolation is a risk. In addition, given the prolonged COVID-19 pandemic with high transmissibility of the SARS-CoV-2, not a few family members may have contracted COVID-19 themselves during the pandemic [31]. Emerging evidence indicates that a SARS-CoV-2 infection, even without hospitalization, can result in long-term psychological symptoms, commonly referred to as the long COVID [32, 33]. Furthermore, the employment status of family members might be compromised, which might not solely be attributable to the impact of a relative's ICU admission but could potentially be influenced by the consequences of COVID-19 [34]. Such uncertainty, confusion, and anxiety experienced in daily life may have heightened the risk of developing PTSD symptoms, and this psychological distress could have been further amplified in the family members of severe COVID-19 ICU patients [4, 12, 35].

Interestingly, this study suggests the possibility that a notable percentage (15%) of family members of COVID-19 patients admitted to the ICU experience delayed-onset PTSD, highlighting the need for ongoing monitoring and support to this population. Delayed-onset PTSD is a widely acknowledged condition, referring to the development of PTSD symptoms at least six months after the traumatic event [36]. The mechanisms behind the development of PTSD in family members during the remote phase following a patient's ICU discharge are not fully understood. One possible explanation is that family members remain focused on the patient's physical recovery during the ICU stay, and the psychological impact of the traumatic experience only starts to emerge once the patient’s condition is stabilized [37]. In addition, several studies have shown that anxiety symptoms in family members during the earlier phase (as indicated by high HADS scores) are predictive of later PTSD symptoms [35, 38], and this study supports that finding. The unique circumstances of the COVID-19 pandemic, including the urgent fear of possibly contracting the virus, social isolation, and financial instability, may have further exacerbated the anxiety of family members after the patient was discharged from the ICU. These observations suggest the psychological distress experienced during the earlier phase influences the development of PTSD symptoms in the remote phase following the patient’s ICU discharge [12].

Overall, these findings emphasize the importance of both early intervention during the ICU stay and long-term family care following a patient’s discharge. Active communication and information sharing with family members is a practical early intervention strategy that can be integrated into routine ICU care [39]. Simply providing information has been shown to be insufficient in preventing psychological symptoms, including PTSD [40]. Bohart et al. concluded that it is essential to equip family members not only with knowledge of the patient’s condition and treatment plan but also with coping skills for the ICU environment, facilitated through active communication with healthcare providers [41]. In addition, offering specific support to help families fulfill their roles as advocates and supporters of the patient is critical [41]. One useful tool for organizing and sharing information may be the ICU diary, which can give family members a sense of control, helping them stay informed about general events and document their support for their loved one, even when direct communication is not possible [42]. On the other hand, several challenges exist in providing long-term family care following a patient's ICU discharge. First, the timely identification of sociodemographic, disease-related, and psychological risk factors is crucial to aid in identifying patients predisposed to an enduring trajectory of post-traumatic stress symptoms, facilitating their inclusion in tailored psychotherapeutic interventions [43]. So far, in addition to the early onset of anxiety symptoms mentioned above, various factors—including being female, younger, having a spousal relationship with the patient, being unemployed, and having pre-existing mental health disorders—have been reported to increase the susceptibility of family members to PICS-F [44, 45]. Indeed, some of these factors, such as being female and having a spousal relationship, align with the findings of this study. Second, it is also crucial to establish a system for long-term follow-up of family members after patients’ ICU discharge. However, there is limited knowledge regarding the optimal ways to support family members of ICU patients following their discharge. PICS follow-up clinics/services are expected to provide care not only for patients but also for their family members [46–49]. Third, clinicians need to be mindful of the long-term psychological symptoms in both COVID-19 patients and their families [10]. This awareness will ultimately contribute to effective prevention and seamless care for both the patients and their families following patients’ discharge from the ICU [50].

This study has several limitations. First, this is a small, single-center study, and the subjects were limited to a key family member (usually a surrogate decision-maker) who agreed to participate in this study. Thus, caution should be exercised in the selection bias and generalizing our results to other family members of COVID-19 patients. Second, the survey started at different timepoints for different subjects, because this study was started more than a year after the COVID-19 pandemic began in Tokyo. However, this eventually contributed to collecting more data over a year after the patient’s ICU discharge. Third, it is crucial to recognize that the psychological symptoms observed in this study were derived from self-reported questionnaire screening and, hence, do not constitute diagnostic results. Moreover, among the various psychological symptoms screened, only PTSD assessed by the IES-R can be attributed to a family member's ICU stay in this survey. This distinction arises, because other questionnaires, such as HADS for anxiety and depression, PSQI for sleep disorders, and SF-12 for HRQOL assessment, do not explicitly establish a direct link between these symptoms and the ICU admission of a family member due to their questionnaire design [31]. IES-R may also be influenced by recall bias, since various experiences before and after the patient’s ICU admission could alter original memories during the ICU admission. Comparing the families of COVID-19 patients who were not admitted to the ICU as a control group could be one way to address this limitation. Consequently, it remains largely unclear whether these symptoms were attributed to the ICU admission of loved ones with COVID-19. Fourth, this study is a retrospective observational descriptive study and does not comprehensively address risk factors for the development of PTSD in family members of severe COVID-19 patients, such as pre-existing mental health conditions, social isolation, or financial instability [18]. Further research is still needed to clarify these aspects.

## Conclusion

The prevalence of PTSD-related symptoms among family members of severely ill COVID-19 patients stayed high up to 18 months after the patients’ ICU discharge. Some showed delayed onset of PTSD-related symptoms in the remote period after the patient’s ICU discharge. This highlights the need for a support system for family members that extends into the remote post-ICU period.

## Data Availability

The data sets generated and/or analyzed during the current study are not publicly available due to permission to share the data has not been obtained from the institutional review board, but are available from the corresponding author on reasonable request.

## Abbreviations

HADS (–A, –D): the hospital anxiety and depression scale (–anxiety, –depression)
HRQOL: Health-related quality of life
ICU: Intensive care unit
IES-R: The impact of event scale-revised
MCS: The mental health component summary
PCS: The physical health component summary
PICS(–F): Post-intensive care syndrome (–family)
PSQI: The Pittsburgh sleep quality index
PTSD: Post-traumatic stress disorder
RCS: The role-social component summary
SF-12: The 12-item short-form health survey
